# Profiling of miRNAs Contained in Circulating Extracellular Vesicles and Associated with Sepsis Development in Burn Patients: A Proof-of-Concept Study

**DOI:** 10.3390/ijms26051844

**Published:** 2025-02-21

**Authors:** Martina Schiavello, Ornella Bosco, Barbara Vizio, Alberto Sciarrillo, Anna Pensa, Emanuele Pivetta, Fulvio Morello, Daniela Risso, Giuseppe Montrucchio, Filippo Mariano, Enrico Lupia

**Affiliations:** 1 Department of Medical Science, University of Turin, 10126 Turin, Italy; martina.schiavello@unito.it (M.S.); ornella.bosco@unito.it (O.B.); barbara.vizio@unito.it (B.V.); emanuele.pivetta@unito.it (E.P.); fulvio.morello@unito.it (F.M.); filippo.mariano@unito.it (F.M.); enrico.lupia@unito.it (E.L.); 2 Plastic Surgery and Burn Center, Department of General and Specialized Surgery, City of Health and Science, CTO Hospital, 10126 Turin, Italy; albertosciarrillo@gmail.com (A.S.); anna.pensa@unito.it (A.P.); drisso@cittadellasalute.to.it (D.R.); 3 Nephrology, Dialysis and Transplantation Unit, City of Health and Science, CTO Hospital, 10126 Turin, Italy

**Keywords:** extracellular vesicles, miRNAs, sepsis, burn patients

## Abstract

Sepsis is the leading cause of mortality in patients with burn injuries and it may represent, in these patients, a real diagnostic challenge. Here we studied the profile of miRNAs contained in extracellular vesicles (EVs) (EV-miRNAs) isolated from plasma from burn patients complicated by sepsis at admission and 7 days later. We enrolled 28 burn patients, 18 with (Burn Septic Patients—BSPs) and 10 without (Burn non-Septic Patients—BnSPs) sepsis. Ten healthy subjects (HSs) were used as additional controls. After EV isolation by charge precipitation and miRNA extraction, we proceeded with a two-phase approach. Through a first screening phase, we identified 178 miRNAs differentially expressed in BSPs compared to HSs. Among these, by a validation phase based on qRT-PCR, we found that miR-483-5p, miR-193a-5p, and miR-188-3p were increased in the BSPs compared to the BnSPs and HSs. Upon ROC analysis, all three miRNAs showed a good accuracy in differentiating BSPs from BnSPs, especially miR-483-5p (AUC = 0.955, *p*-value = 0.001). Moreover, we found 173 miRNAs differentially expressed in BSPs after 7 days from enrollment compared to T0, among whose miR-1-3p, miR-34a-3p, and miR-193a-5p decreased in BSPs after 7 days, in parallel with a decrease in SOFA scores. Finally, the other two miRNAs, miR-34a-3p and miR-193a-5p, positively correlated with the SOFA score. In conclusion, we identified several miRNAs—namely miR-483-5p, miR-193a-5p, and miR-188-3p—with potential clinical utility as diagnostic biomarkers in a heterogeneous population of burn patients at high risk of developing sepsis. Moreover, we found some miRNAs (miR-1-3p, miR-34a-3p, and miR-193a-5p) that vary according to the course of sepsis and others (miR-34a-3p and miR-193a-5p) that are associated with its clinical severity.

## 1. Introduction

In patients with burn injuries caused by serious thermodynamic damage the incidence of sepsis is extremely high [1]. The prognosis for burn patients with sepsis is poor, with estimates of a >60% mortality rate in burn patients resulting from infectious complications [2]. These patients have a higher susceptibility to bacterial invasion and sepsis due to the compromised skin barrier and to the systemic dysregulation and immunosuppression brought about in response to burn injury. For these reasons, an early diagnosis of sepsis is crucial for the management and outcome of critically burned patients.

Several clinical scores have been proposed to help in diagnosing sepsis [3,4]; however, some of these are not appropriate for burn patients [5,6,7]. Moreover, although many biomarkers have been proposed, there is still a lack of reliable indicators that are useful for diagnosis and disease assessment, whereas, due to the complexity of burn patients, it seems wise to find a combination of biomarkers, instead of a single biomarker, that are potentially applicable in these patients [8,9,10].

Extracellular vesicles (EVs) are functional vesicles released by all human cells and found in various body fluids [11]. They act as pivotal vehicles for intercellular communication [12]. EVs are composed of lipid bilayers and carry various cargos, such as lipids, proteins, and miRNAs, to target cells or organs via systemic circulation [11]. The contents of EV cargoes differ significantly depending on the origin and the state of cells [12]. In recent years, the study of EVs has deeply evolved as a consequence of the availability of more sophisticated isolation methods, and EVs have been widely investigated as markers for diseases, including chronic and critical illnesses [13,14,15,16].

miRNAs are small non-coding 18–25 nucleotide RNAs that interfere with the expression of up to 30% of protein-coding genes in mammalian cells [17]. The clinical relevance of miRNAs in sepsis has been previously reported [18,19]. For instance, miR-150 has been shown to be a predictor for survival in patients with critical illness and sepsis [20]. Increased levels of serum miR-133a has also been demonstrated to be an independent predictor for mortality in sepsis patients [21]. Moreover, plasma miR-15 and miR-27a levels were significantly reduced while levels of miR-34a were increased in patients with septic shock [22]. Despite a long road travelled in the study of EVs in several diseases, the nature and function of miRNAs contained in EVs (EV-miRNAs) isolated from plasma from patients with sepsis-associated burn injuries are still unknown.

Here, we conducted a proof-of-concept study aimed at evaluating the utility of EV-miRNAs isolated from plasma from burn patients complicated by sepsis as diagnostic biomarkers for the rapid detection of sepsis. Aiming to establish a comprehensive workflow to evaluate miRNA expression in EVs from plasma from burn patients, the following two-phase approach was used: (i) a preliminary screening phase of EV-miRNAs followed by (ii) a validation phase.

## 2. Results

### 2.1. Patient Characteristics

A total of 28 burn patients were included in this study. Among these, sepsis was present in 18 cases. A total of 10 healthy subjects (HSs) were also enrolled and used as additional controls. The study groups (Burn non-Septic Patients—BnSPs vs. Burn-Septic Patients—BSPs) did not differ in age or gender distribution, as well as in average % total body surface area (TBSA). At 1 month of hospitalization, all 28 patients were alive. Blood cultures were positive in all BSPs. Detailed information on the demographic and clinical characteristics of the patients and controls is summarized in Table 1.

### 2.2. Characterization of EVs Isolated from Plasma from Burn Patients and Healthy Subjects

EVs were isolated via a charge-based precipitation method from plasma samples of 18 BSPs, 10 BnSPs, and 10 HSs. We used Nanoparticle Tracking Analysis (NTA) to determine the size and the concentration of the particles isolated from plasma samples. The diameter of the particles prepared was in the ranges of 100–400 nm in the three study groups (Figure S1), whereas the concentration of particles was 2.12 × 10^10^ in HSs, 5.17 × 10^10^ in BnSPs, and 8.20 × 10^10^ in BSPs. We used flow cytometric analysis to verify the expression of specific EV surface markers, showing that CD9 and CD63, considered specific EV markers, were expressed in particles isolated from plasma samples of the HSs, BnSPs, and BSPs (Figure S2). Therefore, EVs were successfully isolated from plasma from both burn patients and healthy subjects.

### 2.3. Sepsis Significantly Alters the Profile of miRNAs Contained in EVs (EV-miRNAs) Isolated from Plasma from Patients with Burn Injuries

Among 754 miRNAs screened, 178 miRNAs were differentially expressed in the BSPs compared to the HSs. Moreover, 16 miRNAs were exclusively detected in EVs isolated from the BSPs, while 32 miRNAs were specific only to EVs isolated from the HSs (Table S1). Finally, the expression of 528 miRNAs was not detected.

### 2.4. Validation of Differentially Expressed EV-miRNAs in Patients with Burn Injuries Complicated or Not with Sepsis

To validate the results obtained in the preliminary discovery analysis, we enrolled an additional control group represented by patients with burn injuries with no deterioration in SOFA scores > 2 and no blood culture positivity at the enrollment and during the following 72 h of hospitalization (Burn non-Septic Patients—BnSPs).

**Figure 1:**
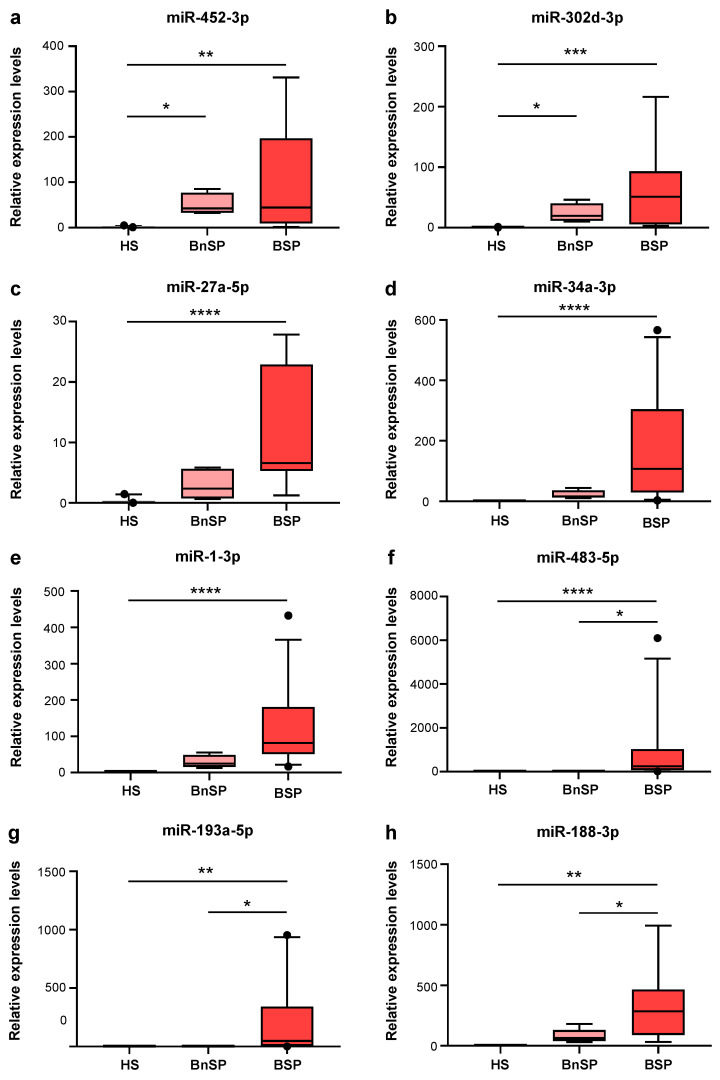
Validation by qRT-PCR of candidate miRNAs contained in plasma EVs. Relative expression levels of miR-452-3p panel (**a**), miR-302d-3p panel (**b**), miR-27a-5p panel (**c**), miR-34a-3p panel (**d**); miR-1-3p panel (**e**); miR-483-5p panel (**f**); miR-193a-5p panel (**g**); miR-188-3p panel (**h**) in EVs isolated from plasma from HSs (*n* = 10), BnSPs (*n* = 10), and BSPs (*n* = 18). Data are presented as median (IQR), normalized with a reference exogenous control (Cel-miR-39). * *p* < 0.05, ** *p* < 0.01, *** *p* < 0.001, **** *p* < 0.0001.

In this validation phase conducted via qRT-PCR, we considered the nine more abundantly expressed EV-miRNAs. Of these, we chose the eight EV-derived miRNAs with the highest levels of expression in the BSPs compared to the HSs: miR-452-3p, miR-27a-5p, miR-302d-3p, miR-483-5p, miR-1-3p, miR-193a-5p, miR-34a-3p, and miR-1255a (Table S1). Moreover, we chose one miRNA expressed only in EVs prepared from BSPs: miR-188-3p (Table S1). The validation analysis of the more abundantly expressed EV-miRNAs found during the screening phase confirmed that several miRNAs—namely miR-452-3p, miR-302d-3p, miR-27a-5p, miR-34a-3p, miR-1-3p, miR-483-5p, and miR-193a-5p—were significantly more expressed in the BSPs compared to the HSs (Figure 1a–g). Moreover, the qRT-PCR validation showed that miR-188-3p was increased in the BSPs, confirming the results obtained in the screening phase, but was detectable in the HSs at low expression levels, at variance from what was observed in the array screening, where no expression was observed (Figure 1h, Table S1). miR-1255a showed no statistically significant trend of up-regulation in the BSPs. Of note, miR-452-3p and miR-302d-3p were found to have increased in the BnSPs compared to the HSs, while miR-483-5p, miR-193a-5p, and miR-188-3p were increased in the BSPs compared to the BnSPs.

### 2.5. Accuracy of Plasma EV-miRNAs in Patients with Burn Injuries for Sepsis Diagnosis

**Figure 2:**
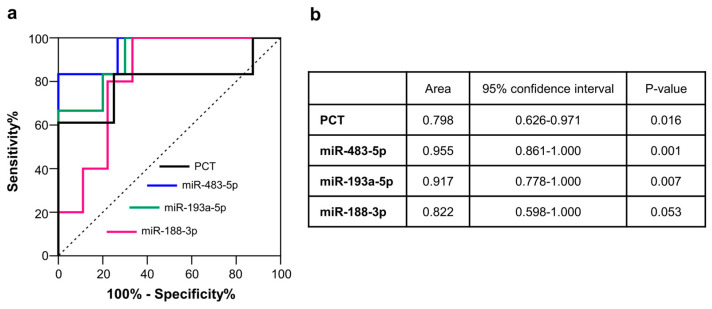
Receiver operating characteristic (ROC) curves of the candidate EV-miRNAs and procalci–tonin (PCT) for distinguishing between BSPs and BnSPs. panel (**a**) Area Under the Curve (AUC); panel (**b**) Discriminatory performance of each EV-miRNA examined.

The diagnostic performance of those EV-miRNAs we found increased in the BSPs compared to the BnSPs (miR-483-5p, miR-193a-5p, and miR-188-3p). Distinguishing features between these two patients groups was evaluated by ROC curve analysis (Figure 2a,b). miR-483-5p showed the best AUC (0.955, *p*-value = 0.001), but miR-193a-5p (0.917, *p*-value 0.007) also showed a good accuracy, whereas miR-188-3p was close to reaching a statistical significance (0.822, *p*-value 0.053). These AUCs were superior to that of procalcitonin (0.798, *p*-value 0.016), a well-established marker for the diagnosis of sepsis [9,10].

### 2.6. Temporal Dynamics of Plasma EV-miRNAs During the Course of Sepsis

In order to characterize the temporal trends of miRNAs-derived from EVs isolated from BSPs, we performed a miRNA screening on EVs isolated from plasma from 5 BSPs at the time of enrollment (T0) and after 7 days (T1).

In these patients, we observed a significant decrease in SOFA scores at T1 compared to T0 (*p* = 0.0004). The clinical characteristics of these BSPs at the different time points are summarized in Table 2.

Among 754 miRNAs screened, 173 miRNAs were differentially expressed in the BSPs at T1 compared to T0. Moreover, 31 miRNAs were exclusively detected in the BSPs at T1, while 6 miRNAs were specific only to BSPs at T0 (Table S2). Finally, the expression of 544 miRNAs was not detected.

**Figure 3:**
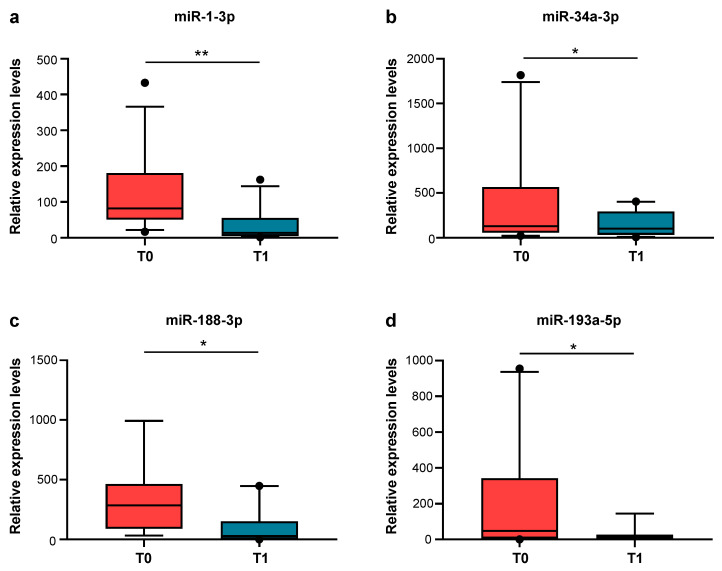
Validation via qRT-PCR of candidate miRNAs contained in EVs isolated from BSPs at T0 and at T1. Relative expression levels of miR-1-3p panel (**a**), miR-34a-3p panel (**b**), miR-188-3p panel (**c**), and miR-193a-5p panel (**d**) in EVs isolated from plasma from BSPs at T0 and at T1. Data are presented as median (IQR), normalized with a reference exogenous control (Cel-miR-39). * *p* < 0.05, ** *p* < 0.01.

The validation analysis carried out via qRT-PCR of the more abundantly expressed EV-derived miRNAs showed that miR-1-3p, miR-34a-3p, miR-188-3p, and miR-193a-5p were more expressed in the BSPs at T0 compared to T1 (Figure 3a–d). miR-483-5p showed a trend of increased expression in the BSPs at T0 compared to T1, but was not statistically significant (Figure S3a). On the contrary, miR-452-3p, miR-302d-3p, and miR-27a-5p did not show any difference in the expression levels at T0 compared to T1.

**Figure 4:**
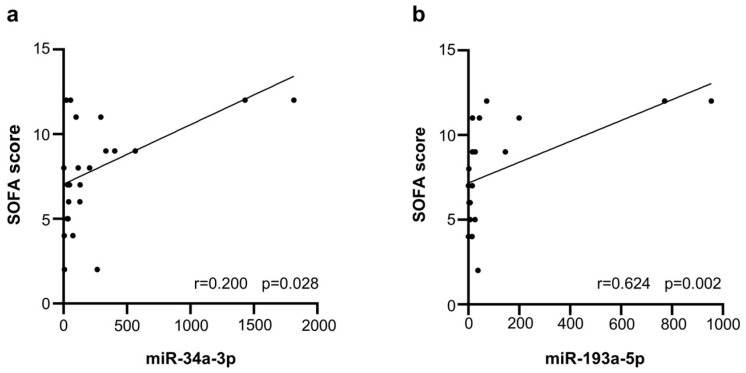
Correlation between EV-miRNA expression levels and SOFA scores in BSPs: panel (**a**) miR-34a-3p; panel (**b**) miR-193a-5p.

We also analyzed the correlation between the SOFA scores, commonly used for the evaluation of the clinical severity of critically ill patients, and selected EV-miRNAs in the BSPs. We found a positive correlation between the highest SOFA score and an increase in miR-34a-3p (r = 0.200, *p* = 0.028), miR-193a-5p (r = 0.624, *p* = 0.002), and miR-483-5p (r = 0.509, *p* = 0.003) expression both at T0 and T1 (Figure 4a,b and Figure S3b). On the contrary, miR-452-3p, miR-302d-3p, and miR-27a-5p were not correlated with SOFA scores.

## 3. Discussion

After a severe burn injury, the body enters a pathological condition of excessive inflammation, hypermetabolism, and acute immune response [23]. Understanding early which patients with burn injuries may develop sepsis could be a major milestone in terms of diagnosis and personalized decision therapies.

Sepsis is the most common complication in severe burn patients and tends to be aggressive, rapid, and fatal [24]. Therefore, the prognosis of sepsis in burn patients depends on accurate diagnosis and timely management [25]. The search for sepsis biomarkers is an exciting and never-ending story [26,27,28]. Recently, the rapid development of multi-level omics biological technologies and computer analytic tools has provided a strong technical foundation for the early warning identification and treatment of the disease [29,30].

In addition to their role in regulating gene expression, circulating miRNAs were proposed as next-generation biomarkers in manifold diseases [31,32]. The diagnostic potential of miRNAs is based on their extraordinary stability and resistance to storage handling [33]. This stability at least partly relies on the inclusion of miRNAs in extracellular vesicles (EVs), where they are protected against degradation or destruction [34].

In our proof-of-concept study, aimed to test the hypothesis that sepsis alters the profile of miRNAs contained in EVs (EV-miRNAs) in burn patients, we used a two-phase approach. In a first set of experiments, we examined circulating EV-miRNA profiles by array technology in patients with burn injury complicated by sepsis (BSPs) in comparison with healthy subjects (HSs). We found that, among 754 miRNAs screened, 178 miRNAs were differentially expressed in the BSPs compared to the HSs. Moreover, 16 miRNAs were exclusively detected in EVs isolated from the BSPs, while 32 miRNAs were specific only to EVs isolated from the HSs. The number of samples in the screening analysis was limited to a pool of five patients, but we further verified the results of the array investigation via qRT-PCR analysis, which was performed on all the patients enrolled in the study. In this set of validation experiments, we performed the analysis in the BSPs, the HSs, and, additionally, in a population of patients with burn injury without evidence of infection (BnSPs). Thus, we identified a pool of miRNAs potentially useful for the diagnosis of sepsis in a heterogeneous population at high risk of developing sepsis like burn patients. We found that eight miRNAs (miR-452-3p, miR-302d-3p, miR-27a-5p, miR-34a-3p, miR-1-3p, miR-483-5p, miR-193a-5p, and miR-188-3p) were more expressed in the BSPs compared to the HSs. Furthermore, two miRNAs (miR-452-3p, miR-302d-3p) were more expressed in the BnSPs compared to the HSs, suggesting a potential association of these miRNAs with burn injuries. Of note, three miRNAs (miR-483-5p, miR-193a-5p, and miR-188-3p) were found to have had a higher expression in BSPs compared to BnSPs, suggesting their potential value for the diagnosis of sepsis in burn patients. All three of these miRNAs showed a good accuracy in discriminating BSPs from BnSPs. This suggests that implementing the use of miRNAs together with the currently used markers, such as procalcitonin (PCT), may be better than the use of a single marker, as has already been established in other diseases [35,36].

Increased expression of selected miRNAs has also been observed in various cancers [37], cardiovascular diseases [38], and in sepsis [18]. In particular, miR-483-5p levels were found to be upregulated in the lung tissues of sepsis-induced acute lung injury (ALI) murine models as compared to the corresponding control groups [39]. The study authors demonstrated that pro-inflammatory cytokines, namely IL-1b and IL-6, were down-regulated in mice with sepsis-induced ALI by miR-483-5p knockdown. Similarly, serum miR-483-5p has been proven to be a potential prognostic marker in septic patients in a prospective observational study [40], which is consistent with our results.

Previous research has shown that exosomal miR-193a-5p, together with other miRNAs, predicts the survival of septic patients with high confidence [41].

miR-188-3p is an independent prognostic factor in colorectal cancer [42] and is expressed in atherosclerosis [43]. To the best of our knowledge, no data referring to miR-188-3p are reported to be associated with sepsis and therefore it could be proposed as a novel early warning marker of burns-associated sepsis in the future.

When we look at miRNA expression in a later phase of the disease, when the clinical conditions of BSPs were improving (BSP T1) compared to the time of enrollment (BSP T0), we found that, among the 754 miRNAs screened, 173 were differentially expressed. Moreover, 31 miRNAs were exclusively detected in BSPs at T1, while 6 were specific only to BSPs at T0. In addition, we validated the levels of the most expressed miRNAs and we found that the levels of four miRNAs (miR-1-3p, miR-34a-3p, miR-188-3p, and miR-193a-5p) decreased after 7 days (BSP T1), paralleling the decrease in SOFA scores and the improved clinical conditions of these patients. Interestingly, miR-34a-3p and miR-193a-5p showed a positive correlation with SOFA scores, suggesting that these miRNAs may be proposed as potential markers for assessing disease severity and that combining SOFA scores with specific miRNAs can increase sensitivity when prognosticating the outcome of sepsis.

In a previous study by Chen et al., miR-34a expression was shown to increase in rats with LPS-induced sepsis [44]. In contrast, other authors revealed that the serum expression level of miR-34a-5p was significantly decreased in patients with neonatal sepsis compared with healthy neonates [45]. This discrepancy in the increased and decreased expression of miR-34a in sepsis might be due to the -3p and -5p strands that define the fate of cellular processes as observed in cell proliferation, migration, and invasion in chronic diseases like cervical cancer [46].

Our study presents some advantages compared to others available in the literature. For instance, at variance from other studies that enrolled only healthy controls, our study included an additional control group comprising patients with burn injuries but no evidence of infection or sepsis. In addition, there was no significant difference in %TBSA between the BSPs and BnSPs, making the two groups similar for this important variable. Distinguishing sepsis patients from healthy subjects is easier than distinguishing patients with burn injuries who develop sepsis from those who did not. Therefore, the results from our study are more convincing than those of other studies that exclusively enrolled normal healthy controls.

On the other hand, our study has several limitations. First, the sample size was relatively small, and thus additional multi-center studies are required to confirm our findings. Moreover, we evaluated BSPs only at two different time points, whereas a better evaluation conducted at several time-points would have been useful to improve the evaluation of the proposed EV-miRNAs. Third, it would be of interest to evaluate the same miRNAs in a population of septic patients with different etiology to establish the universal potential value of selected miRNAs for the diagnosis and prognostic stratification of patients with sepsis. Finally, in our study, we did not explore whether and how the EV-miRNAs we identified as differentially expressed may act in terms of mediators of the pathological mechanisms involved in sepsis development and/or progression in burn patients. Further studies will be needed in order to provide mechanistic information on these topics.

Despite these limitations, our results suggest that circulating EV-miRNAs may be proposed as reliable biomarkers in burn patients to differentiate those with sepsis from those without infection. Moreover, EV-miRNAs seem to have a prominent association with the course of sepsis in burn patients. Future developments will include the study of the functional role of EV-miRNAs here identified, with the perspective of proposing future innovative therapeutic approaches useful for the treatment of burn patients complicated by sepsis.

## 4. Materials and Methods

### 4.1. Patients and Sample Collection

In this prospective observational study, we recruited 40 patients with Total Body Surface Areas (TBSAs) comprised between 10% and 50%. Of these, 18 burn-septic patients (BSPs) and 10 burn non-septic patients (BnSPs) were considered eligible and enrolled in the study. Patient enrollment was conducted at the Burn Center of the “Città della Salute e della Scienza di Torino” University Hospital—CTO Site from November 2022 to January 2024. A total of 12 patients were excluded and considered non-eligible because they did not fit with the inclusion and exclusion criteria as follows.

The inclusion criteria for burn patients were the following: ≥18 years of age, a TBSA >10% and <50%, and informed consent for participation in the study. The exclusion criteria included the following: age < 18 years, a TBSA < 10% and >50%, cancer (active or recent history), autoimmune or chronic inflammatory disease, and HBV/HCV/HIV/SARS-CoV-2 infection. Burn patients admitted to the Burn Center were regularly screened for the insurgence of sepsis, which was diagnosed according to the criteria defined by the Sepsis-3 guidelines [3], modified as follows: an increase in the Sequential Organ Failure Assessment (SOFA) score of ≥2 points plus blood culture positivity. The severity of organ dysfunction was estimated using the SOFA score [47]. Patients with burn injuries with no deterioration in SOFA score > 2 and no blood culture positivity at the enrollment and during the following 72 h of hospitalization were classified as Burn non-Septic Patients (BnSPs) and used as controls.

A total of 10 healthy subjects (HS) without comorbidities and paired for sex and age were also enrolled and used to constitute an additional control group.

Written informed consent was acquired from all enrolled patients and healthy volunteers.

We collected the following data concerning the demographic and clinical characteristics of the study patients: age, gender, comorbidities, total body surface burned area (%TBSA), mean arterial pressure (MAP), Sequential Organ Failure Assessments (SOFA) score, and laboratory findings.

The study was approved by the Institutional Ethics and Review Board of the “Citta della Salute e della Scienza di Torino” University Hospital, Turin, Italy (n. CS2/815) and conducted according to the ethical standards set out by the Declaration of Helsinki and its later amendments.

For patients and healthy subjects, whole blood was collected from a central venous catheter or from a peripheral vein via clean venipuncture using a 21-gauge infusion set, respectively, in EDTA tubes (BD Vacutainer, Reading, UK), and processed within 1 h. The blood samples were first centrifuged at 1600× *g* for 10 min at 4 °C, then the plasma was collected and stored at −80 °C until EV isolation and subsequent analyses were carried out.

### 4.2. Study Design

**Figure 5:**
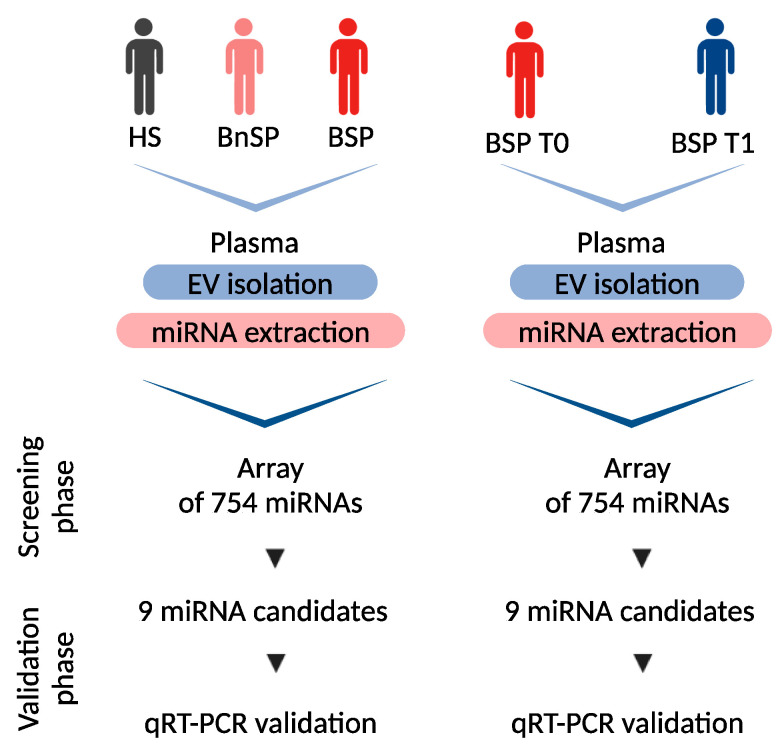
Study design. Created with http://biorender.com/, accessed on 30 December 2024.

This study was designed in two phases (Figure 5): (i) the screening phase and (ii) the validation phase.

For the screening phase, two pooled samples of miRNAs derived from EVs isolated from plasma (EV-miRNAs) prepared from HSs (*n* = 5) and BSPs (*n* = 5) were screened using a miRNA array card. Each group was composed of a mix of EV-miRNAs from five individuals paired for sex and age. For all samples, EV isolation and miRNA extraction from EVs were performed separately and mixed only after EV-miRNA extraction.

For the validation phase, EV-miRNAs from individual samples of HSs (*n* = 10), BnSPs (*n* = 10), BSPs (*n* = 18) were analyzed via qRT-PCR for the expression of the nine more abundantly expressed EV-derived miRNAs identified during the screening phase.

Moreover, we studied the temporal trend of expression of selected EV-miRNAs by two additional phases: (i) screening by miRNA array card of two pooled samples composed of EV-miRNAs prepared from five BSPs at the time of enrollment (T0, *n* = 5) and after 7 days (T1, *n* = 5); (ii) validation phase: analysis via qRT-PCR of the EV-miRNAs from individual samples prepared from BSPs at T0 (*n* = 18) and at T1 (*n* = 18) for the expression of the nine more abundantly expressed EV-derived miRNAs identified during the screening phase.

### 4.3. Isolation of EVs from Plasma via the Charge-Based Precipitation Method

EVs were isolated using the charge-based precipitation method from plasma samples of 18 BSPs, 10 BnSPs, and 10 HSs.

An amount of 1 mL of plasma samples from patients and healthy subjects, stored at −80 °C, were centrifugated at 5000× *g* at 4 °C for 30 min to remove cellular debris and platelet contamination. EVs were isolated via the charge-based precipitation method, as previously described [48]. Briefly, a protamine (P) (Sigma, St. Louis, MO, USA)/Polyethylene glycol (PEG 35,000; Merck KGaA, Darmstadt, Germany) precipitation solution (P/PEG; Sigma, St. Louis, MO, USA) (0.2 g PEG 35,000 and 1 mg protamine chloride/mL; 1:4) was added to the plasma samples. After overnight incubation at 4 °C, the mixture was centrifugated at 1500× *g* for 30 min at 4 °C, and then a second centrifugation was performed at 1500× *g* for 10 min at 4 °C. Finally, the pellet was re-suspended in 150 µL of PBS-free particles and stored at −80° C for subsequent RNA extraction.

### 4.4. EV Characterization

#### 4.4.1. Nanoparticle Tracking Analysis (NTA)

The particles isolated as described above were characterized following the recommendations set out in “Minimal Information for Studies of Extracellular Vesicles” (MISEV) 2023 [49]. To characterize particles isolated from plasma in terms of quantity and size distribution, we used Nanoparticle Tracking Analysis (NTA) using a NanoSight LM10 instrument equipped with 405 nm laser and a Nanosight Tracking Analyses 2.3 Analytical Software (Malvern Panalytical Ltd., Malvern, UK). Briefly, EVs were diluted in particle-free PBS before our analysis was carried out (1:1000). Three consequent 30-s records per sample were acquired. The minimum expected particle size, minimum track length, and biomedical light unit setting were set to automatic, and the detection threshold was set to 4 to reveal all the particles.

#### 4.4.2. Flow Cytometry

In order to investigate the EV expression of specific surface markers by flow cytometry, 20 µL of EVs were labelled with either a phycoerythrin (PE; Invitrogen, ThermoFisher Scientific, Carlsbad, CA, USA)-conjugated mouse anti-human IgG1 monoclonal antibody against the tetraspanins CD9 (clone eBioSN4, C3-3A2) or PE-Cyanine7 (Invitrogen, ThermoFisher Scientific)-conjugated mouse anti-human IgG1 monoclonal antibody against the tetraspanins CD63 (clone H5C6) at a volume of 5 µL/test in a final volume of 100 µL of diluted particle-free PBS, for 1 h at room temperature in the dark. Mouse IgG1 kappa isotype control monoclonal antibodies lacking specificity for CD9 or CD63 (P3.6.2.8.1, PE or PE-Cyanine7, Invitrogen, ThermoFisher Scientific) were used to measure the signal from non-specific flow cytometry interactions. The Attune NxT Small Particle Side-Scatter Filter (488/10) (Invitrogen, ThermoFisher Scientific) was installed to enable SSC resolution at the scale required to visualize nanoparticles in the Attune NxT Acoustic Focusing Flow Cytometer system (ThermoFisher Scientific).

For fluorochrome-conjugated antibody compensation, the AbC Total Antibody Compensation Bead Kit (Life Technologies, ThermoFisher Scientific) was used. For the established gating strategies of EV samples, we used Flow Cytometry Sub-Micron Particle Size Reference Kit (Life Technologies, ThermoFisher Scientific), which provides a set of green fluorescent microsphere suspensions (with a nominal diameter of 0.1, 0.2, 0.5, and 1.0 µm) to serve as reliable size references for flow cytometry, according to the minimal information for the standardized reporting of extracellular vesicles flow cytometry experiments (MIFlowCyt-EV) [50].

The acquisition was performed at 100 µL of the total draw volume and 25 µL/min. Set options were set on 20,000 events. Control experiments for flow cytometric characterization were performed with a buffer control, an isotype control, and the single staining of EVs characterization markers (CD9 and CD63). Data were analyzed with the Attune NxT 3.2 Software.

### 4.5. RNA Extraction from Plasma EVs

miRNAs were isolated from 150 µL of EV pellet using the miRNeasy mini kit (Qiagen, Crawley, UK) according to the manufacturer’s instructions. Spike-in control miRNA-39 (Applied Biosystem, ThermoFisher Scientific) from Caenorhabditis elegans solution (1.6 × 10^8^ copies/µL) was added as an exogenous control for RNA extraction efficiency.

High-quality RNA, primarily miRNAs and other small RNA, was collected in the final elution volume of 20 µL of RNase-free water and stored at −80 °C.

### 4.6. Reverse Transcription

An amount of 2 µL of individual miRNA extracts was reverse-transcribed into complementary DNA (cDNA) using the TaqMan Advanced miRNA cDNA Synthesis Kit (Applied Biosystem, ThermoFisher Scientific) according to the manufacturer’s instructions. Briefly, 5 µL of reverse transcription product were used for pre-amplification using a custom miR-Amp Primer Mix and the miR-Amp Master Mix (Applied Biosystem, ThermoFisher Scientific). Finally, 50 µL of pre-amplified cDNA were stored at −20 °C until further analysis for up to 2 months.

### 4.7. TaqMan Array

In the screening phase, pre-amplification cDNA samples were mixed into pools derived from five individual patient samples from BSPs and HSs in a final volume of 20 µL. qRT-PCR was set up using the TaqMan Fast Advanced Master Mix (Applied Biosystem, ThermoFisher Scientific) and pre-amplified cDNA pools from BSPs and HSs were diluted 1:10 in 0.1× TE buffer.

The expression profile of a panel of 754 human miRNAs was evaluated by TaqMan array human microRNA A + B cards (ThermoFisher Scientific) by loading each fill reservoir with 100 µL of the sample-specific PCR reaction mix and using the QuantStudio 12K Flex Real-Time PCR System (Applied Biosystem, ThermoFisher Scientific). Only the miRNAs found to be expressed in both replicates were considered.

Connect Data Analysis Apps (ThermoFisher Scientific) was used to calculate Raw C_t_ values, with an automatic baseline and threshold and to calculate RQ (2^−ddC^_t_) values. C_t_ values > 40 or with Amp score < 0.7 were excluded from the analysis.

### 4.8. qRT-PCR

In the validation phase, we performed single-tube assays on individual samples from each patient enrolled in the study using a QuantStudio 1 Real-Time PCR System (Applied Biosystem, ThermoFisher Scientific). Pre-amplified cDNA samples were diluted 1:10 in 0.1X TE buffer. Briefly, mastermix, assays, water, and diluted pre-amplified cDNA samples were placed in individual wells of an optical 96-well reaction plate. Each reaction was performed in triplicate. The utilized primers are listed in Table S3. Samples were run using the following parameters: 50 °C 2 min, 95 °C 10 min, 40 cycles of 95 °C 15 s and 60 °C 1 min, and a 4 °C hold.

The relative miRNA expression was calculated using the comparative cycle threshold (2^−ddCt^) method normalized to cel-miR-39 levels (exogenous controls).

All calculations were performed using the QuantStudio Real-Time PCR Software v1.3 (Applied Biosystem, ThermoFisher Scientific).

### 4.9. Statistical Analysis

Descriptive statistics were reported as median (interquartile range, IQR) or mean (±standard error of mean, SEM), according to data distribution as assessed via the Shapiro–Wilk test. Comparison between groups was carried out via a Kruskal–Wallis one-way analysis of variance on ranks followed by Dunn’s multiple comparison tests, or unpaired or paired Student’s *t*-test, as appropriate. The diagnostic accuracy of each miRNA was calculated as the area under the receiver operating characteristic curve (AUC–ROC).

A *p*-value < 0.05 was considered significant.

Data were collected using an Excel spreadsheet (Microsoft Office 365 ProPlus) and analyses were conducted using the GraphPad Prism 9.0 software for Windows and Macintosh (GraphPad Software, La Jolla, CA, USA).

## 5. Conclusions

Our results suggest the potential utility of several circulating EV-miRNAs, namely miR-483-5p, -miR-193a-5p, and -miR-188-3p, as biomarkers for the diagnosis of sepsis in a heterogeneous population of burn patients at high risk of developing sepsis. Furthermore, we identified other EV-miRNAs, such as miR-34a-3p and miR-193a-5p, which are associated with the clinical severity of sepsis, whereas others (miR-1-3p, miR-34a-3p, and miR-193a-5p) vary according to the course of sepsis. In the future, these novel circulating biomarkers might serve as promising tools for the diagnosis of sepsis in burn patients and could aid in the prognostic stratification of these patients. In addition, our proof-of-concept findings establish an important step forward in developing our understanding of EV-miRNA’s pathophysiology and of their potential contribution in ameliorating sepsis diagnosis.

## Figures and Tables

**Table 1:** Demographic and clinical characteristics of patients with burn injuries and healthy subjects.

Characteristics	Healthy Subjects (HSs) (*n* = 10)	Burn Non-Septic Patients (BnSPs) (*n* = 10)	Burn-Septic Patients (BSPs) (*n* = 18)	*p*-Value
Age, year, mean ± SD	57.60 (±13.28)	52.43 (±18.43)	58.07 (±16.41)	0.659
Gender ratio (M/F)	5/5	6/4	11/7	0.317
Comorbidities				
Diabetes Mellitus, n (%)	0	0	2	0.117
Hypertension, n (%)	0	0	8	<0.0001
% TBSA	-	20.00 (10.00–35.00)	30.00 (19.00–41.50)	0.121
MAP	-	80.00 (43.00–83.00)	81.50 (33.00–100.00)	0.329
SOFA score	-	0	10.39 (±2.35)	<0.0001
Laboratory findings				
White Blood Cell 10^9/^L	4.60 (4.35–5.28)	6.99 (5.73–12.24)	12.24 (11.00–17.78) ****°	<0.0001
Hemoglobin g/dL	13.55 (13.28–13.85)	12.90 (11.93–14.25)	8.85 (8.57–9.57) ****°°	<0.0001
Platelets 10^9/^L	261.0 (±26.44)	218.6 (±45.10)	136.3 (±62.00) ****°°°	<0.0001
Creatinine mg/dL	0.933 (±0.06)	1.14 (±0.81)	1.67 (±0.99)	0.221
Procalcitonin ng/mL	-	0.39 (0.35–0.88)	2.52 (0.15–19.68)	0.021
Lactate mmol/L	-	0.80 (0.50–1.20)	1.40 (1.10–1.80)	0.018
Bilirubin mg/dL	-	0.80 (0.55–1.35)	1.20 (0.60–1.70)	0.368
NT-pro-BNP ng/L	-	157.00 (27.00–500.00)	1528 (143.00–44,552)	0.003
Troponin I ng/L	-	4.50 (±1.80)	28.00 (6.00–303.00)	0.005
Myoglobin µg/L	-	79.00 (±50.76)	128.00 (41.00–14,265)	0.227

% TBSA, Total Body Surface Area; MAP, mean arterial pressure; SOFA, Sequential Organ Failure Assessment; mean (±SD) or median (IQR) as appropriate. **** vs. HSs, *p* < 0.0001; ° vs. BnSPs, *p* < 0.05; °° vs. BnSPs, *p* < 0.01; °°° vs. BnSPs, *p* < 0.001.

**Table 2:** Patients Characteristics.

Characteristics	Burn-Septic Patients BSP T0 (*n* = 18)	Burn-Septic Patients BSP T1 (*n* = 18)	*p*-Value
MAP	81.50 (33.00–100.00)	78.00 (60.00–98.00)	0.170
P/F	286.3 (±106.9)	277.3 (±81.81)	0.948
SOFA score	10.39 (±2.35)	7.33 (±3.51)	<0.001
Laboratory findings			
White Blood Cell 10^9/^L	12.24 (11.00–17.78)	12.71 (6.39–35.53)	0.502
Hemoglobin g/dL	8.85 (8.57–9.57)	9.05 (7.70–11.20)	0.412
Platelets 10^9/^L	136.3 (±62.00)	344.00 (±127.5)	<0.001
Creatinine mg/dL	1.53 (0.86–3.80)	0.77 (0.30–2.35)	0.003
Procalcitonin ng/mL	2.52 (0.15–99.67)	0.56 (0.16–5.86)	<0.001
Lactate mmol/L	1.40 (0.10–1.80)	1.05 (0.40–2.00)	0.017
Bilirubin mg/dL	1.20 (0.60–1.70)	0.85 (0.30–8.70)	0.734
NT-pro-BNP ng/L	1528 (143.00–44,552)	686.00 (37.00–9622)	0.065
Copeptin pmol/L	42.80 (3.00–167.3)	28.90 (6.40–493.00)	0.464
Troponin I ng/L	28.00 (6.00–303.00)	27.00 (5.00–520.00)	0.452
Myoglobin µg/L	128.00 (41.00–14,265)	63.00 (34.00–503.00)	0.006

SOFA, Sequential Organ Failure Assessment; MAP, mean arterial pressure; P/F, PaO_2_/FiO_2_; mean (±SD) or median (IQR) as appropriate.

## Data Availability

Deidentified participant data will be made available on a collaborative basis upon reasonable request. Data and research materials used in this study are available upon request to qualified researchers for purposes of replication, further analysis, and academic collaboration.

## Supplementary Materials

The following supporting information can be downloaded at: https://www.mdpi.com/article/10.3390/ijms26051844/s1.
