# Supplementary material for: Profiling of miRNAs Contained in Circulating Extracellular Vesicles and Associated with Sepsis Development in Burn Patients: A Proof-of-Concept Study
Source: Int J Mol Sci. 2025 Feb 21;26(5):1844. doi: 10.3390/ijms26051844 (PMC11899136; doi:10.3390/ijms26051844)
Supplement: Supplementary file 1 [file ijms-26-01844-s001.zip › ijms-3428915-supplementary.pdf]

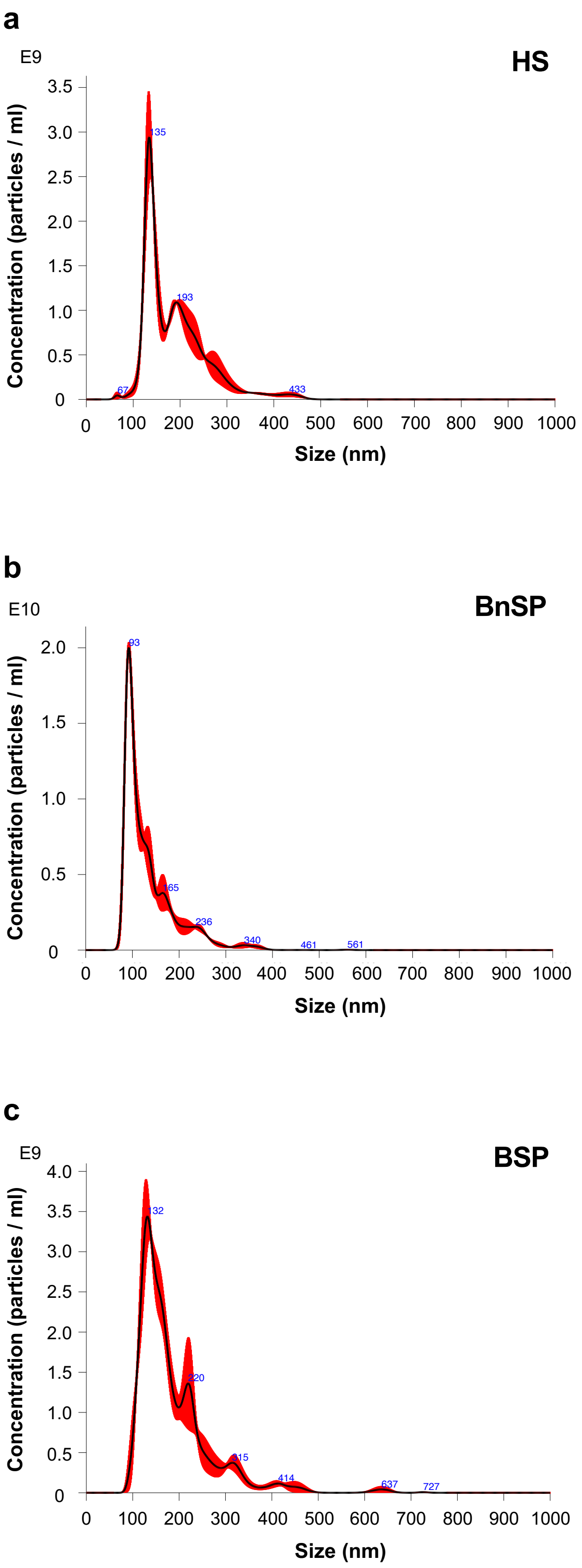

Figure S1. Nanosight EV characterization. Representative images of NTA analysis referred to (**a**) healthy subjects (HS), (**b**) burn non septic patients (BnSP), (**c**) burn septic patients (BSP).

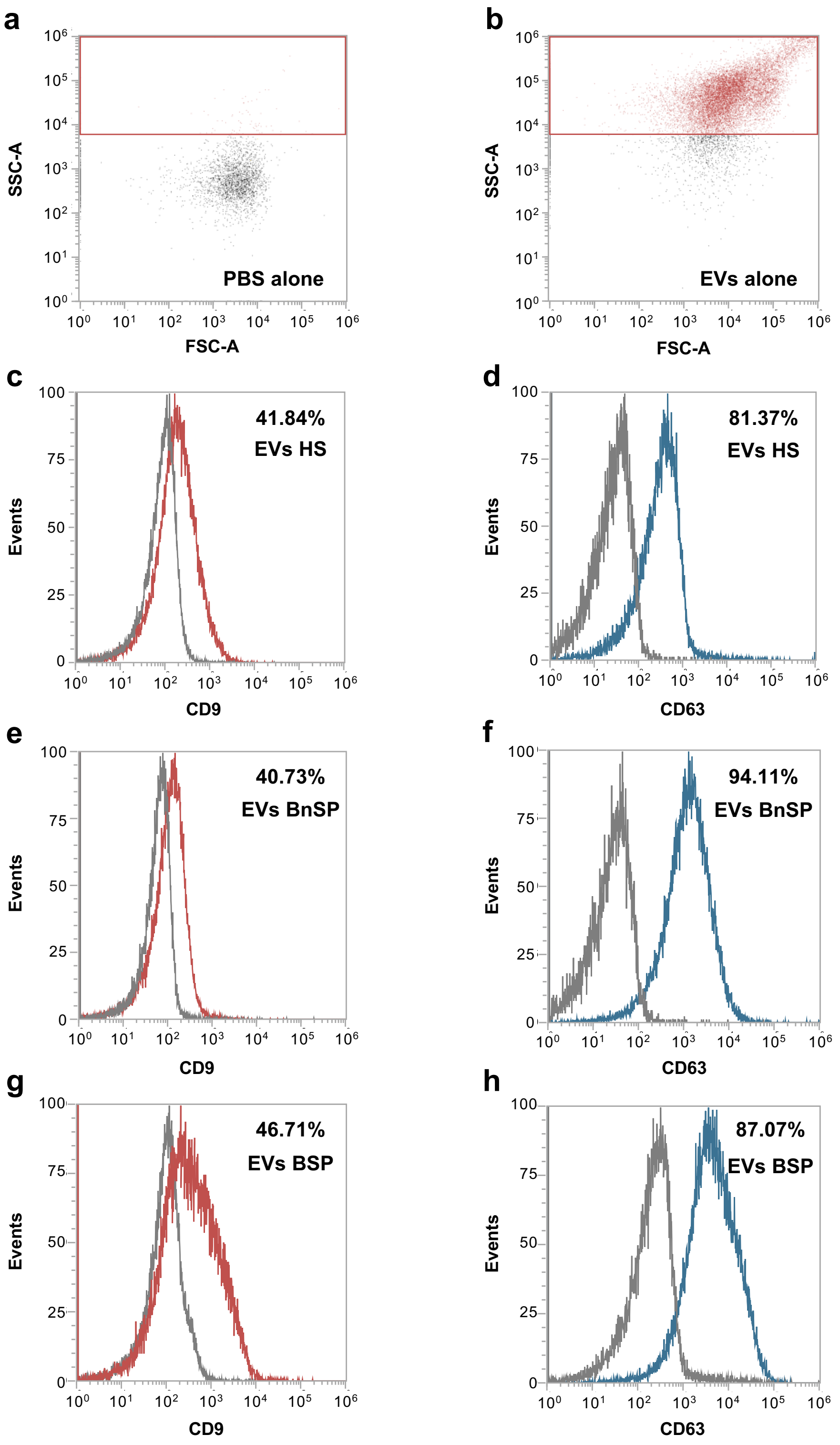

Figure S2. Detection and characterization of extracellular vesicles (EVs) by flow cytometry. Morphological representative experiment using EVs for gaiting strategy with (**a**) free-particle PBS and (**b**) EV samples. EVs were immunostained against the tetrapanin CD9 (red) and CD63 (blu) and compared with their appropriate isotype control (grey). The percentage represente the overlay between CD and isotype control. The graphs is representative of Evs from Healthy Subjects (HS, **c** and **d**), Burn non Septic Patients (BnSP, **e** and **f)** and Burn Septic Patients (BSP, **g** and **h**).

Supplementary Table S1

Sample ID	miRNA ID	Mean Equivalent Cq	Sample ID	Mean Equivalent Cq	Rq vs HS
HS	hsa-miR-495-3p-478136_mir	28.540	BSP	33.200	0,286
HS	hsa-miR-499a-5p-478139_mir	32.254	BSP	36.852	0,299
HS	hsa-miR-451a-478107_mir	17.336	BSP	21.455	0,417
HS	hsa-miR-485-3p-478125_mir	28.666	BSP	32.716	0,437
HS	hsa-miR-491-5p-478132_mir	30.669	BSP	34.663	0,455
HS	hsa-miR-101-3p-477863_mir	26.734	BSP	29.998	0,753
HS	hsa-miR-148b-3p-477824_mir	27.902	BSP	31.043	0,820
HS	hsa-miR-127-3p-477889_mir	29.322	BSP	32.178	1,000
HS	hsa-miR-409-3p-478084_mir	29.341	BSP	31.907	1,180
HS	hsa-miR-342-3p-478043_mir	25.996	BSP	28.481	1,293
HS	hsa-miR-183-3p-477936_mir	34.885	BSP	37.177	1,426
HS	hsa-miR-185-5p-477939_mir	25.344	BSP	27.582	1,535
HS	hsa-miR-16-5p-477860_mir	22.561	BSP	24.751	1,562
HS	hsa-miR-486-5p-478128_mir	18.788	BSP	20.99	1,574
HS	hsa-miR-191-5p-477952_mir	23.028	BSP	25.184	1,624
HS	hsa-miR-150-5p-477918_mir	21.472	BSP	23.604	1,651
HS	hsa-miR-330-3p-478030_mir	28.914	BSP	30.965	1,747
HS	hsa-miR-15a-5p-477858_mir	22.08	BSP	24.036	1,866
HS	hsa-miR-664a-3p-478193_mir	25.863	BSP	27.621	2,066
HS	hsa-miR-26b-5p-478418_mir	23.172	BSP	24.979	2,070
HS	hsa-miR-25-3p-477994_mir	22.832	BSP	24.547	2,206
HS	hsa-miR-28-3p-477999_mir	27.575	BSP	29.242	2,281
HS	hsa-miR-543-478155_mir	27.373	BSP	28.969	2,311
HS	hsa-miR-10b-5p-478494_mir	32.177	BSP	33.691	2,536
HS	hsa-miR-16-2-3p-477931_mir	27.37	BSP	28.754	2,677
HS	hsa-miR-494-3p-478135_mir	30.793	BSP	32.224	2,685
HS	hsa-miR-532-5p-478151_mir	30.08	BSP	31.478	2,748
HS	hsa-miR-17-5p-478447_mir	25.535	BSP	26.928	2,757
HS	hsa-miR-181c-5p-477934_mir	28.977	BSP	30.358	2,779
HS	hsa-miR-140-3p-477908_mir	28.721	BSP	30.049	2,884
HS	hsa-miR-126-3p-477887_mir	22.273	BSP	23.582	2,922
HS	hsa-miR-128-3p-477892_mir	27.967	BSP	29.27	2,933
HS	hsa-let-7b-5p-478576_mir	28.27	BSP	29.53	3,024
HS	hsa-miR-93-5p-478210_mir	27.084	BSP	28.314	3,087
HS	hsa-miR-424-5p-478092_mir	26.411	BSP	27.633	3,103
HS	hsa-miR-106b-3p-477866_mir	30.26	BSP	31.418	3,130
HS	hsa-miR-26a-5p-477995_mir	23.594	BSP	24.799	3,139
HS	hsa-miR-18a-5p-478551_mir	28.158	BSP	29.300	3,281
HS	hsa-let-7g-5p-478580_mir	27.460	BSP	28.594	3,299
HS	hsa-miR-425-5p-478094_mir	27.894	BSP	28.992	3,383
HS	hsa-miR-126-5p-477888_mir	24.927	BSP	25.808	3,795
HS	hsa-miR-500a-5p-478309_mir	29.242	BSP	30.172	3,798
HS	hsa-miR-27a-3p-478384_mir	28.747	BSP	29.643	3,893
HS	hsa-miR-186-5p-477940_mir	25.838	BSP	26.715	3,940

HS	hsa-miR-100-5p-478224_mir	30.855	BSP	31.712	3,996
HS	hsa-miR-92a-3p-477827_mir	20.476	BSP	21.311	4,058
HS	hsa-miR-339-3p-478325_mir	26.971	BSP	27.693	4,388
HS	hsa-miR-181a-5p-477857_mir	26.205	BSP	26.899	4,474
HS	hsa-miR-23b-5p-477991_mir	30.305	BSP	30.909	4,595
HS	hsa-miR-151a-3p-477919_mir	27.224	BSP	27.824	4,609
HS	hsa-miR-660-5p-478192_mir	30.246	BSP	30.861	4,729
HS	hsa-miR-29c-5p-478005_mir	33.882	BSP	34.405	4,861
HS	hsa-miR-107-478254_mir	27.268	BSP	27.832	4,900
HS	hsa-miR-323a-3p-477853_mir	31.472	BSP	31.986	5,071
HS	hsa-miR-324-5p-478024_mir	29.370	BSP	29.85	5,192
HS	hsa-miR-20a-5p-478586_mir	25.677	BSP	26.142	5,244
HS	hsa-miR-224-5p-477986_mir	29.839	BSP	30.285	5,314
HS	hsa-miR-424-3p-478091_mir	28.899	BSP	29.227	5,564
HS	hsa-miR-625-3p-478179_mir	30.362	BSP	30.677	5,613
HS	hsa-miR-223-3p-477983_mir	23.265	BSP	23.596	5,755
HS	hsa-let-7d-5p-478439_mir	24.399	BSP	24.721	5,792
HS	hsa-miR-23a-3p-478532_mir	22.562	BSP	22.778	6,233
HS	hsa-miR-425-3p-478093_mir	26.885	BSP	27.028	6,328
HS	hsa-miR-188-5p-477943_mir	31.115	BSP	31.240	6,408
HS	hsa-miR-181d-5p-479517_mir	30.272	BSP	30.353	6,842
HS	hsa-miR-374b-5p-478389_mir	27.226	BSP	27.286	6,943
HS	hsa-miR-145-5p-477916_mir	25.016	BSP	24.980	7,421
HS	hsa-miR-505-3p-478145_mir	28.815	BSP	28.779	7,425
HS	hsa-miR-92b-3p-477823_mir	18.926	BSP	18.873	7,515
HS	hsa-miR-429-477849_mir	33.203	BSP	33.138	7,575
HS	a-miR-199a-3p_hsa-miR-199b-3p-477961_m	26.011	BSP	25.94	7,605
HS	hsa-miR-155-5p-477927_mir	31.402	BSP	31.291	7,818
HS	hsa-miR-19a-3p-479228_mir	26.561	BSP	26.440	7,876
HS	hsa-miR-125b-5p-477885_mir	25.736	BSP	25.583	8,052
HS	hsa-miR-338-3p-478037_mir	31.603	BSP	31.435	8,135
HS	hsa-miR-374a-5p-478238_mir	29.463	BSP	29.282	8,204
HS	hsa-miR-181c-3p-477933_mir	34.095	BSP	33.856	8,242
HS	hsa-miR-144-3p-477913_mir	22.898	BSP	22.635	8,381
HS	hsa-miR-1260a-478476_mir	20.665	BSP	20.372	8,561
HS	hsa-miR-99b-5p-478343_mir	26.712	BSP	26.453	8,662
HS	hsa-miR-151a-5p-478505_mir	25.084	BSP	24.718	8,999
HS	hsa-miR-376a-3p-478240_mir	28.214	BSP	27.824	9,489
HS	hsa-miR-484-478308_mir	23.937	BSP	23.482	9,924
HS	hsa-miR-194-5p-477956_mir	29.038	BSP	28.573	9,994
HS	hsa-miR-222-3p-477982_mir	28.579	BSP	28.109	10,028
HS	hsa-miR-125a-5p-477884_mir	25.373	BSP	24.893	10,097
HS	hsa-miR-548a-3p-478157_mir	30.276	BSP	29.793	10,121
HS	hsa-let-7d-3p-477848_mir	23.054	BSP	22.519	10,122
HS	hsa-miR-339-5p-478040_mir	28.546	BSP	28.003	10,551
HS	hsa-miR-130b-3p-477840_mir	27.118	BSP	26.555	10,702
HS	hsa-miR-181a-3p-479405_mir	29.258	BSP	28.637	10,739
HS	hsa-miR-433-3p-478102_mir	27.083	BSP	26.503	10,828

HS	hsa-miR-2110-477971_mir	27.427	BSP	26.825	10,992
HS	hsa-miR-103a-3p-478253_mir	27.4	BSP	26.783	11,104
HS	hsa-miR-30a-3p-478273_mir	30.838	BSP	30.139	11,336
HS	hsa-miR-382-5p-478078_mir	30.764	BSP	30.115	11,350
HS	hsa-miR-24-3p-477992_mir	26.788	BSP	26.134	11,393
HS	hsa-miR-374a-3p-478855_mir	33.666	BSP	32.894	11,928
HS	hsa-miR-1180-3p-477869_mir	29.786	BSP	28.989	12,140
HS	hsa-miR-21-5p-477975_mir	23.094	BSP	22.328	12,313
HS	hsa-miR-598-3p-478172_mir	31.82	BSP	31.037	12,453
HS	hsa-miR-362-5p-478059_mir	32.409	BSP	31.616	12,540
HS	hsa-miR-146a-5p-478399_mir	24.924	BSP	24.091	12,898
HS	hsa-miR-500a-3p-478951_mir	27.997	BSP	27.110	12,924
HS	hsa-miR-361-3p-478055_mir	29.541	BSP	28.644	13,005
HS	hsa-miR-27b-3p-478270_mir	28.136	BSP	27.252	13,360
HS	hsa-miR-361-5p-478056_mir	27.933	BSP	27.047	13,377
HS	hsa-miR-21-3p-477973_mir	28.393	BSP	27.446	13,468
HS	hsa-miR-148a-3p-477814_mir	27.072	BSP	26.173	13,494
HS	hsa-miR-652-3p-478189_mir	26.934	BSP	26.031	13,534
HS	hsa-miR-485-5p-478126_mir	29.996	BSP	29.003	14,417
HS	hsa-miR-885-5p-478207_mir	27.949	BSP	26.857	15,434
HS	hsa-miR-383-5p-478079_mir	35.341	BSP	34.21	15,864
HS	hsa-miR-337-5p-478036_mir	32.57	BSP	31.428	15,972
HS	hsa-miR-204-5p-478491_mir	32.895	BSP	31.74	16,124
HS	hsa-miR-22-3p-477985_mir	23.372	BSP	22.2	16,311
HS	hsa-miR-221-3p-477981_mir	22.387	BSP	21.169	16,847
HS	hsa-miR-134-5p-477901_mir	29.79	BSP	28.535	17,277
HS	hsa-miR-1301-3p-477897_mir	25.129	BSP	23.804	17,510
HS	hsa-miR-423-5p-478090_mir	21.972	BSP	20.686	17,656
HS	hsa-miR-564-478161_mir	32.701	BSP	31.348	17,842
HS	hsa-miR-583-479065_mir	32.218	BSP	30.846	18,088
HS	hsa-miR-941-479217_mir	29.303	BSP	27.929	18,106
HS	hsa-miR-130a-3p-477851_mir	25.601	BSP	24.261	18,320
HS	hsa-miR-200a-3p-478490_mir	26.652	BSP	25.303	18,444
HS	hsa-miR-625-5p-479469_mir	29.061	BSP	27.630	19,525
HS	hsa-miR-320a-478594_mir	21.398	BSP	19.959	19,626
HS	hsa-miR-744-5p-478200_mir	27.901	BSP	26.307	21,853
HS	hsa-miR-320b-478588_mir	23.009	BSP	21.28	23,151
HS	hsa-miR-302b-3p-478591_mir	34.904	BSP	33.187	23,806
HS	hsa-miR-326-478027_mir	26.261	BSP	24.489	24,715
HS	hsa-miR-210-3p-477970_mir	29.493	BSP	27.684	25,375
HS	hsa-miR-125a-3p-477883_mir	31.047	BSP	29.231	25,490
HS	hsa-miR-524-3p-479338_mir	28.239	BSP	26.329	26,239
HS	hsa-miR-628-3p-478181_mir	22.951	BSP	20.979	27,415
HS	hsa-miR-328-3p-478028_mir	26.072	BSP	24.151	27,419
HS	hsa-miR-525-3p-478995_mir	27.56	BSP	25.588	28,405
HS	hsa-miR-10b-3p-477868_mir	30.459	BSP	28.395	29,205
HS	hsa-miR-593-3p-479076_mir	28.55	BSP	26.416	30,659
HS	hsa-miR-448-478105_mir	27.846	BSP	25.754	30,875

HS	hsa-miR-208b-3p-477806_mir	31.159	BSP	29.017	31,965
HS	hsa-miR-503-5p-478143_mir	32.206	BSP	30.020	32,945
HS	hsa-miR-380-3p-477854_mir	28.635	BSP	26.445	33,034
HS	hsa-miR-505-5p-478957_mir	31.594	BSP	29.326	33,660
HS	hsa-miR-22-5p-477987_mir	30.167	BSP	27.822	35,483
HS	hsa-miR-423-3p-478327_mir	26.503	BSP	24.102	38,237
HS	hsa-miR-629-5p-478183_mir	30.355	BSP	27.906	39,530
HS	hsa-miR-378a-3p-478349_mir	26.895	BSP	24.372	40,156
HS	hsa-miR-452-5p-478109_mir	32.597	BSP	30.114	40,458
HS	hsa-miR-502-3p-478348_mir	23.909	BSP	21.348	42,724
HS	hsa-miR-603-479084_mir	34.718	BSP	32.086	43,311
HS	hsa-miR-153-3p-477922_mir	30.823	BSP	28.215	44,139
HS	hsa-miR-18a-3p-477944_mir	30.82	BSP	28.073	46,890
HS	hsa-miR-214-3p-477974_mir	28.722	BSP	25.998	47,827
HS	hsa-miR-663b-479146_mir	24.206	BSP	21.426	47,972
HS	hsa-miR-133a-3p-478511_mir	29.336	BSP	26.565	49,446
HS	hsa-miR-584-5p-478167_mir	22.096	BSP	19.248	50,290
HS	hsa-miR-190a-5p-478358_mir	29.308	BSP	26.471	51,732
HS	hsa-miR-152-3p-477921_mir	27.626	BSP	24.760	52,758
HS	hsa-miR-181b-5p-478583_mir	29.501	BSP	26.591	54,403
HS	hsa-miR-653-5p-479134_mir	26.623	BSP	23.687	55,407
HS	hsa-miR-552-3p-479036_mir	31.08	BSP	28.059	56,672
HS	hsa-miR-645-478188_mir	29.520	BSP	26.497	56,784
HS	hsa-miR-490-3p-478131_mir	23.024	BSP	19.968	60,201
HS	hsa-miR-122-5p-477855_mir	26.108	BSP	23.044	60,548
HS	hsa-miR-548e-3p-478362_mir	31.255	BSP	28.027	65,425
HS	hsa-miR-133b-480871_mir	32.515	BSP	29.309	66,777
HS	hsa-miR-325-478025_mir	28.173	BSP	24.922	68,912
HS	hsa-miR-455-3p-478112_mir	33.736	BSP	30.328	76,847
HS	hsa-miR-562-479047_mir	33.300	BSP	29.774	80,494
HS	**hsa-miR-452-3p-478917_mir**	35.433	BSP	31.841	**84,227**
HS	**hsa-miR-27a-5p-477998_mir**	32.410	BSP	28.776	**86,698**
HS	**hsa-miR-302d-3p-478237_mir**	35.322	BSP	31.685	**86,877**
HS	**hsa-miR-483-5p-478432_mir**	27.313	BSP	23.476	**103,421**
HS	**hsa-miR-1-3p-477820_mir**	23.639	BSP	19.302	**146,257**
HS	**hsa-miR-193a-5p-477954_mir**	28.946	BSP	24.264	**185,794**
HS	**hsa-miR-34a-3p-478047_mir**	36.784	BSP	30.723	**466,273**
HS	**hsa-miR-1255a-478661_mir**	34.043	BSP	27.562	**623,977**
HS	hsa-let-7b-3p-478221_mir	27.175	BSP	-	-
HS	hsa-let-7f-5p-478578_mir	25.484	BSP	-	-
HS	hsa-let-7i-3p-477862_mir	30.612	BSP	-	-
HS	hsa-miR-103a-2-5p-477864_mir	27.283	BSP	-	-
HS	hsa-miR-1249-3p-478654_mir	26.405	BSP	-	-
HS	hsa-miR-130b-5p-477899_mir	27.354	BSP	-	-
HS	hsa-miR-132-3p-477900_mir	26.414	BSP	-	-
HS	hsa-miR-142-3p-477910_mir	26.847	BSP	-	-
HS	hsa-miR-143-3p-477912_mir	26.030	BSP	-	-
HS	hsa-miR-151b-477811_mir	22.894	BSP	-	-

HS	hsa-miR-154-3p-478725_mir	30.561	BSP	-	-
HS	hsa-miR-154-5p-477925_mir	32.164	BSP	-	-
HS	hsa-miR-193b-3p-478314_mir	28.743	BSP	-	-
HS	hsa-miR-20b-5p-477804_mir	26.436	BSP	-	-
HS	hsa-miR-216a-5p-477976_mir	28.771	BSP	-	-
HS	hsa-miR-296-5p-477836_mir	28.023	BSP	-	-
HS	hsa-miR-29a-3p-478587_mir	26.297	BSP	-	-
HS	hsa-miR-29b-3p-478369_mir	28.174	BSP	-	-
HS	hsa-miR-301a-3p-477815_mir	29.972	BSP	-	-
HS	hsa-miR-30b-5p-478007_mir	23.169	BSP	-	-
HS	hsa-miR-30c-5p-478008_mir	23.611	BSP	-	-
HS	hsa-miR-31-5p-478015_mir	29.062	BSP	-	-
HS	hsa-miR-345-5p-478366_mir	28.997	BSP	-	-
HS	hsa-miR-369-3p-478067_mir	28.786	BSP	-	-
HS	hsa-miR-374b-3p-479421_mir	35.145	BSP	-	-
HS	hsa-miR-432-5p-478101_mir	27.615	BSP	-	-
HS	hsa-miR-487b-3p-477835_mir	27.568	BSP	-	-
HS	hsa-miR-497-5p-478138_mir	29.314	BSP	-	-
HS	hsa-miR-518e-3p-479408_mir	32.903	BSP	-	-
HS	hsa-miR-574-3p-478163_mir	29.618	BSP	-	-
HS	hsa-miR-576-3p-478164_mir	25.626	BSP	-	-
HS	hsa-miR-606-479087_mir	28.136	BSP	-	-
HS	hsa-let-7a-5p-478575_mir	-	BSP	23.956	-
HS	hsa-miR-1290-477895_mir	-	BSP	29.199	-
HS	hsa-miR-1292-5p-478691_mir	-	BSP	29.833	-
HS	hsa-miR-15b-5p-478313_mir	-	BSP	24.974	-
HS	**hsa-miR-188-3p-477942_mir**	-	BSP	**28.663**	-
HS	hsa-miR-206-477968_mir	-	BSP	31.788	-
HS	hsa-miR-223-5p-477984_mir	-	BSP	30.502	-
HS	hsa-miR-29b-2-5p-478003_mir	-	BSP	31.442	-
HS	hsa-miR-30d-5p-478606_mir	-	BSP	26.184	-
HS	hsa-miR-377-3p-478075_mir	-	BSP	33.420	-
HS	hsa-miR-501-5p-478142_mir	-	BSP	31.097	-
HS	hsa-miR-548d-5p-480870_mir	-	BSP	31.786	-
HS	hsa-miR-548j-5p-479022_mir	-	BSP	39.639	-
HS	hsa-miR-633-479115_mir	-	BSP	29.653	-
HS	hsa-miR-937-3p-479212_mir	-	BSP	29.465	-
HS	hsa-miR-99b-3p-478216_mir	-	BSP	28.280	

Supplementary Table S2

Sample ID	Target Name	Mean Equivalent Cq	Sample ID	Mean Equivalent Cq	Rq *vs.* HS	Sample ID	Mean Equivalent Cq	Rq *vs.* HS
HS	hsa-miR-495-3p-478136_mir	28.54	BSP T0	33.2	0,286	BSP T1	28.67	0.717
HS	hsa-miR-499a-5p-478139_mir	32.254	BSP T0	36.852	0,299	BSP T1	32.989	0.472
HS	hsa-miR-451a-478107_mir	17.336	BSP T0	21.455	0,417	BSP T1	21.146	0.056
HS	hsa-miR-485-3p-478125_mir	28.666	BSP T0	32.716	0,437	BSP T1	27.731	1.501
HS	hsa-miR-491-5p-478132_mir	30.669	BSP T0	34.663	0,455	BSP T1	28.143	4.523
HS	hsa-miR-101-3p-477863_mir	26.734	BSP T0	29.998	0,753	BSP T1	27.265	0.543
HS	hsa-miR-148b-3p-477824_mir	27.902	BSP T0	31.043	0,820	BSP T1	26.558	1.992
HS	hsa-miR-127-3p-477889_mir	29.322	BSP T0	32.178	1,000	BSP T1	26.105	7.302
HS	hsa-miR-409-3p-478084_mir	29.341	BSP T0	31.907	1,180	BSP T1	25.962	8.063
HS	hsa-miR-342-3p-478043_mir	25.996	BSP T0	28.481	1,293	BSP T1	26.298	0.637
HS	hsa-miR-185-5p-477939_mir	25.344	BSP T0	27.582	1,535	BSP T1	24.4	1.511
HS	hsa-miR-16-5p-477860_mir	22.561	BSP T0	24.751	1,562	BSP T1	22.953	0.595
HS	hsa-miR-486-5p-478128_mir	18.788	BSP T0	20.99	1,574	BSP T1	19.852	0.376
HS	hsa-miR-191-5p-477952_mir	23.028	BSP T0	25.184	1,624	BSP T1	21.976	1.628
HS	hsa-miR-150-5p-477918_mir	21.472	BSP T0	23.604	1,651	BSP T1	24.09	0.128
HS	hsa-miR-330-3p-478030_mir	28.914	BSP T0	30.965	1,747	BSP T1	25.525	8.227
HS	hsa-miR-15a-5p-477858_mir	22.08	BSP T0	24.036	1,866	BSP T1	23.046	0.402
HS	hsa-miR-664a-3p-478193_mir	25.863	BSP T0	27.621	2,066	BSP T1	26.34	0.557
HS	hsa-miR-26b-5p-478418_mir	23.172	BSP T0	24.979	2,070	BSP T1	22.215	1.525
HS	hsa-miR-25-3p-477994_mir	22.832	BSP T0	24.547	2,206	BSP T1	23.058	0.672
HS	hsa-miR-28-3p-477999_mir	27.575	BSP T0	29.242	2,281	BSP T1	25.539	3.222
HS	hsa-miR-543-478155_mir	27.373	BSP T0	28.969	2,311	BSP T1	25.737	2.408
HS	hsa-miR-16-2-3p-477931_mir	27.37	BSP T0	28.754	2,677	BSP T1	27.53	0.694
HS	hsa-miR-494-3p-478135_mir	30.793	BSP T0	32.224	2,685	BSP T1	29.925	1.433
HS	hsa-miR-532-5p-478151_mir	30.08	BSP T0	31.478	2,748	BSP T1	29.986	0.838
HS	hsa-miR-17-5p-478447_mir	25.535	BSP T0	26.928	2,757	BSP T1	24.107	2.113
HS	hsa-miR-181c-5p-477934_mir	28.977	BSP T0	30.358	2,779	BSP T1	27.805	1.77
HS	hsa-miR-140-3p-477908_mir	28.721	BSP T0	30.049	2,884	BSP T1	27.114	2.392
HS	hsa-miR-126-3p-477887_mir	22.273	BSP T0	23.582	2,922	BSP T1	21.365	1.473
HS	hsa-miR-128-3p-477892_mir	27.967	BSP T0	29.27	2,933	BSP T1	26.636	1.975
HS	hsa-let-7b-5p-478576_mir	28.27	BSP T0	29.53	3,024	BSP T1	27.096	1.772
HS	hsa-miR-93-5p-478210_mir	27.084	BSP T0	28.314	3,087	BSP T1	25.614	2.176
HS	hsa-miR-424-5p-478092_mir	26.411	BSP T0	27.633	3,103	BSP T1	27.003	0.521
HS	hsa-miR-106b-3p-477866_mir	30.26	BSP T0	31.418	3,130	BSP T1	27.644	4.751
HS	hsa-miR-26a-5p-477995_mir	23.594	BSP T0	24.799	3,139	BSP T1	21.976	2.41
HS	hsa-miR-18a-5p-478551_mir	28.158	BSP T0	29.3	3,281	BSP T1	25.861	3.858
HS	hsa-let-7g-5p-478580_mir	27.46	BSP T0	28.594	3,299	BSP T1	25.91	2.298
HS	hsa-miR-425-5p-478094_mir	27.894	BSP T0	28.992	3,383	BSP T1	26.508	2.052
HS	hsa-miR-126-5p-477888_mir	24.927	BSP T0	25.808	3,795	BSP T1	23.894	1.585
HS	hsa-miR-500a-5p-478309_mir	29.242	BSP T0	30.172	3,798	BSP T1	29.572	0.625
HS	hsa-miR-27a-3p-478384_mir	28.747	BSP T0	29.643	3,893	BSP T1	24.617	13.749
HS	hsa-miR-186-5p-477940_mir	25.838	BSP T0	26.715	3,940	BSP T1	24.527	1.948
HS	hsa-miR-100-5p-478224_mir	30.855	BSP T0	31.712	3,996	BSP T1	31.445	0.522
HS	hsa-miR-92a-3p-477827_mir	20.476	BSP T0	21.311	4,058	BSP T1	19.561	1.481

HS	hsa-miR-339-3p-478325_mir	26.971	BSP T0	27.693	4,388	BSP T1	25.315	2.473
HS	hsa-miR-107-478254_mir	27.268	BSP T0	27.832	4,900	BSP T1	25.55	2.583
HS	hsa-miR-382-5p-478078_mir	30.764	BSP T0	30.115	11,350	BSP T1	26.82	12.089
HS	hsa-miR-1180-3p-477869_mir	29.786	BSP T0	28.989	12,140	BSP T1	28.926	1.406
HS	hsa-miR-362-5p-478059_mir	32.409	BSP T0	31.616	12,540	BSP T1	28.578	11.168
HS	hsa-miR-27b-3p-478270_mir	28.136	BSP T0	27.252	13,360	BSP T1	25.682	4.302
HS	hsa-miR-1301-3p-477897_mir	25.129	BSP T0	23.804	17,510	BSP T1	21.842	7.566
HS	hsa-miR-130a-3p-477851_mir	25.601	BSP T0	24.261	18,320	BSP T1	22.954	4.918
HS	hsa-miR-125a-3p-477883_mir	31.047	BSP T0	29.231	25,490	BSP T1	26.617	16.93
HS	hsa-miR-505-5p-478957_mir	31.594	BSP T0	29.326	33,660	BSP T1	26.18	33.032
HS	hsa-miR-629-5p-478183_mir	30.355	BSP T0	27.906	39,530	BSP T1	26.72	9.757
HS	hsa-miR-18a-3p-477944_mir	30.82	BSP T0	28.073	46,890	BSP T1	26.995	10.977
HS	hsa-miR-584-5p-478167_mir	22.096	BSP T0	19.248	50,290	BSP T1	18.387	10.131
HS	hsa-miR-181a-5p-477857_mir	26.205	BSP T0	26.899	4.474	BSP T1	24.225	3.097
HS	hsa-miR-23b-5p-477991_mir	30.305	BSP T0	30.909	4.595	BSP T1	27.753	4.542
HS	hsa-miR-151a-3p-477919_mir	27.224	BSP T0	27.824	4.609	BSP T1	25.08	3.425
HS	hsa-miR-660-5p-478192_mir	30.246	BSP T0	30.861	4.729	BSP T1	30.832	0.523
HS	hsa-miR-29c-5p-478005_mir	33.882	BSP T0	34.405	4.861	BSP T1	30.953	5.899
HS	hsa-miR-323a-3p-477853_mir	31.472	BSP T0	31.986	5.071	BSP T1	29.74	2.609
HS	hsa-miR-324-5p-478024_mir	29.37	BSP T0	29.85	5.192	BSP T1	26.497	5.753
HS	hsa-miR-20a-5p-478586_mir	25.677	BSP T0	26.142	5.244	BSP T1	24.226	2.147
HS	hsa-miR-224-5p-477986_mir	29.839	BSP T0	30.285	5.314	BSP T1	27.968	2.872
HS	hsa-miR-424-3p-478091_mir	28.899	BSP T0	29.227	5.564	BSP T1	28.547	0.989
HS	hsa-miR-625-3p-478179_mir	30.362	BSP T0	30.677	5.613	BSP T1	27.514	5.578
HS	hsa-miR-223-3p-477983_mir	23.265	BSP T0	23.596	5.755	BSP T1	21.397	2.865
HS	hsa-let-7d-5p-478439_mir	24.399	BSP T0	24.721	5.792	BSP T1	22.654	2.632
HS	hsa-miR-23a-3p-478532_mir	22.562	BSP T0	22.778	6.233	BSP T1	20.498	3.284
HS	hsa-miR-425-3p-478093_mir	26.885	BSP T0	27.028	6.328	BSP T1	24.14	5.193
HS	hsa-miR-181d-5p-479517_mir	30.272	BSP T0	30.353	6.842	BSP T1	27.607	4.979
HS	hsa-miR-374b-5p-478389_mir	27.226	BSP T0	27.286	6.943	BSP T1	25.967	1.879
HS	hsa-miR-145-5p-477916_mir	25.016	BSP T0	24.98	7.421	BSP T1	22.131	5.798
HS	hsa-miR-505-3p-478145_mir	28.815	BSP T0	28.779	7.425	BSP T1	25.949	5.727
HS	hsa-miR-92b-3p-477823_mir	18.926	BSP T0	18.873	7.515	BSP T1	17.737	1.79
HS	hsa-miR-429-477849_mir	33.203	BSP T0	33.138	7.575	BSP T1	38.593	0.019
HS	199a-3p_hsa-miR-199b-3p-47	26.011	BSP T0	25.94	7.605	BSP T1	23.263	5.275
HS	hsa-miR-155-5p-477927_mir	31.402	BSP T0	31.291	7.818	BSP T1	29.58	2.776
HS	hsa-miR-19a-3p-479228_mir	26.561	BSP T0	26.44	7.876	BSP T1	23.923	4.89
HS	hsa-miR-125b-5p-477885_mir	25.736	BSP T0	25.583	8.052	BSP T1	25.789	0.757
HS	hsa-miR-338-3p-478037_mir	31.603	BSP T0	31.435	8.135	BSP T1	30.939	1.244
HS	hsa-miR-374a-5p-478238_mir	29.463	BSP T0	29.282	8.204	BSP T1	28.859	1.193
HS	hsa-miR-181c-3p-477933_mir	34.095	BSP T0	33.856	8.242	BSP T1	30.035	12.921
HS	hsa-miR-144-3p-477913_mir	22.898	BSP T0	22.635	8.381	BSP T1	23.169	0.642
HS	hsa-miR-1260a-478476_mir	20.665	BSP T0	20.372	8.561	BSP T1	20.188	1.079
HS	hsa-miR-99b-5p-478343_mir	26.712	BSP T0	26.453	8.662	BSP T1	24.147	4.645
HS	hsa-miR-151a-5p-478505_mir	25.084	BSP T0	24.718	8.999	BSP T1	23.255	2.753
HS	hsa-miR-376a-3p-478240_mir	28.214	BSP T0	27.824	9.489	BSP T1	28.117	0.84
HS	hsa-miR-484-478308_mir	23.937	BSP T0	23.482	9.924	BSP T1	21.184	5.291
HS	hsa-miR-194-5p-477956_mir	29.038	BSP T0	28.573	9.994	BSP T1	28.509	1.133

HS	hsa-miR-222-3p-477982_mir	28.579	BSP T0	28.109	10.028	BSP T1	26.252	3.94
HS	hsa-miR-125a-5p-477884_mir	25.373	BSP T0	24.893	10.097	BSP T1	22.496	5.769
HS	hsa-miR-548a-3p-478157_mir	30.276	BSP T0	29.793	10.121	BSP T1	28.874	2.075
HS	hsa-let-7d-3p-477848_mir	23.054	BSP T0	22.519	10.122	BSP T1	21.015	3.184
HS	hsa-miR-339-5p-478040_mir	28.546	BSP T0	28.003	10.551	BSP T1	24.771	10.75
HS	hsa-miR-130b-3p-477840_mir	27.118	BSP T0	26.555	10.702	BSP T1	23.711	8.334
HS	hsa-miR-181a-3p-479405_mir	29.258	BSP T0	28.637	10.739	BSP T1	27.1	3.456
HS	hsa-miR-433-3p-478102_mir	27.083	BSP T0	26.503	10.828	BSP T1	23.974	6.778
HS	hsa-miR-2110-477971_mir	27.427	BSP T0	26.825	10.992	BSP T1	25.225	3.614
HS	hsa-miR-103a-3p-478253_mir	27.4	BSP T0	26.783	11.104	BSP T1	24.227	7.078
HS	hsa-miR-30a-3p-478273_mir	30.838	BSP T0	30.139	11.336	BSP T1	32.559	0.235
HS	hsa-miR-24-3p-477992_mir	26.788	BSP T0	26.134	11.393	BSP T1	23.405	8.191
HS	hsa-miR-374a-3p-478855_mir	33.666	BSP T0	32.894	11.928	BSP T1	33.848	0.683
HS	hsa-miR-21-5p-477975_mir	23.094	BSP T0	22.328	12.313	BSP T1	21.287	2.747
HS	hsa-miR-598-3p-478172_mir	31.82	BSP T0	31.037	12.453	BSP T1	28.569	7.472
HS	hsa-miR-146a-5p-478399_mir	24.924	BSP T0	24.091	12.898	BSP T1	22.271	4.941
HS	hsa-miR-500a-3p-478951_mir	27.997	BSP T0	27.11	12.924	BSP T1	26.59	2.055
HS	hsa-miR-361-3p-478055_mir	29.541	BSP T0	28.644	13.005	BSP T1	26.26	7.531
HS	hsa-miR-361-5p-478056_mir	27.933	BSP T0	27.047	13.377	BSP T1	25.34	4.739
HS	hsa-miR-21-3p-477973_mir	28.393	BSP T0	27.446	13.468	BSP T1	27.165	1.815
HS	hsa-miR-148a-3p-477814_mir	27.072	BSP T0	26.173	13.494	BSP T1	23.659	8.364
HS	hsa-miR-652-3p-478189_mir	26.934	BSP T0	26.031	13.534	BSP T1	23.524	8.344
HS	hsa-miR-485-5p-478126_mir	29.996	BSP T0	29.003	14.417	BSP T1	26.428	9.311
HS	hsa-miR-885-5p-478207_mir	27.949	BSP T0	26.857	15.434	BSP T1	27.186	1.333
HS	hsa-miR-337-5p-478036_mir	32.57	BSP T0	31.428	15.972	BSP T1	28.726	11.277
HS	hsa-miR-204-5p-478491_mir	32.895	BSP T0	31.74	16.124	BSP T1	31.898	1.567
HS	hsa-miR-22-3p-477985_mir	23.372	BSP T0	22.2	16.311	BSP T1	21.367	3.151
HS	hsa-miR-221-3p-477981_mir	22.387	BSP T0	21.169	16.847	BSP T1	19.743	4.911
HS	hsa-miR-134-5p-477901_mir	29.79	BSP T0	28.535	17.277	BSP T1	26.041	10.554
HS	hsa-miR-423-5p-478090_mir	21.972	BSP T0	20.686	17.656	BSP T1	18.881	6.69
HS	hsa-miR-564-478161_mir	32.701	BSP T0	31.348	17.842	BSP T1	30.345	3.966
HS	hsa-miR-583-479065_mir	32.218	BSP T0	30.846	18.088	BSP T1	30.166	3.213
HS	hsa-miR-941-479217_mir	29.303	BSP T0	27.929	18.106	BSP T1	26.877	4.164
HS	hsa-miR-200a-3p-478490_mir	26.652	BSP T0	25.303	18.444	BSP T1	25.502	1.743
HS	hsa-miR-625-5p-479469_mir	29.061	BSP T0	27.63	19.525	BSP T1	25.022	12.906
HS	hsa-miR-320a-478594_mir	21.398	BSP T0	19.959	19.626	BSP T1	18.73	4.99
HS	hsa-miR-744-5p-478200_mir	27.901	BSP T0	26.307	21.853	BSP T1	22.582	31.35
HS	hsa-miR-320b-478588_mir	23.009	BSP T0	21.28	23.151	BSP T1	24.216	0.336
HS	hsa-miR-302b-3p-478591_mir	34.904	BSP T0	33.187	23.806	BSP T1	33.119	2.706
HS	hsa-miR-326-478027_mir	26.261	BSP T0	24.489	24.715	BSP T1	22.756	8.915
HS	hsa-miR-210-3p-477970_mir	29.493	BSP T0	27.684	25.375	BSP T1	26.34	6.983
HS	hsa-miR-524-3p-479338_mir	28.239	BSP T0	26.329	26.239	BSP T1	26.73	2.204
HS	hsa-miR-628-3p-478181_mir	22.951	BSP T0	20.979	27.415	BSP T1	21.662	1.893
HS	hsa-miR-328-3p-478028_mir	26.072	BSP T0	24.151	27.419	BSP T1	21.571	17.777
HS	hsa-miR-525-3p-478995_mir	27.56	BSP T0	25.588	28.405	BSP T1	26.405	1.749
HS	hsa-miR-593-3p-479076_mir	28.55	BSP T0	26.416	30.659	BSP T1	26.325	3.622
HS	hsa-miR-448-478105_mir	27.846	BSP T0	25.754	30.875	BSP T1	26.777	1.647
HS	hsa-miR-208b-3p-477806_mir	31.159	BSP T0	29.017	31.965	BSP T1	30.481	1.257

HS	hsa-miR-503-5p-478143_mir	32.206	BSP T0	30.02	32.945	BSP T1	29.091	6.8
HS	hsa-miR-380-3p-477854_mir	28.635	BSP T0	26.445	33.034	BSP T1	26.648	3.113
HS	hsa-miR-22-5p-477987_mir	30.167	BSP T0	27.822	35.483	BSP T1	25.909	14.815
HS	hsa-miR-423-3p-478327_mir	26.503	BSP T0	24.102	38.237	BSP T1	22.143	16.125
HS	hsa-miR-378a-3p-478349_mir	26.895	BSP T0	24.372	40.156	BSP T1	25.091	2.704
HS	hsa-miR-452-5p-478109_mir	32.597	BSP T0	30.114	40.458	BSP T1	28.824	10.734
HS	hsa-miR-502-3p-478348_mir	23.909	BSP T0	21.348	42.724	BSP T1	28.377	0.035
HS	hsa-miR-603-479084_mir	34.718	BSP T0	32.086	43.311	BSP T1	32.551	3.48
HS	hsa-miR-153-3p-477922_mir	30.823	BSP T0	28.215	44.139	BSP T1	28.218	4.777
HS	hsa-miR-214-3p-477974_mir	28.722	BSP T0	25.998	47.827	BSP T1	27.078	2.453
HS	hsa-miR-663b-479146_mir	24.206	BSP T0	21.426	47.972	BSP T1	22.925	1.883
HS	hsa-miR-133a-3p-478511_mir	29.336	BSP T0	26.565	49.446	BSP T1	27.534	2.738
HS	hsa-miR-190a-5p-478358_mir	29.308	BSP T0	26.471	51.732	BSP T1	26.883	4.217
HS	hsa-miR-152-3p-477921_mir	27.626	BSP T0	24.76	52.758	BSP T1	23.543	13.308
HS	hsa-miR-181b-5p-478583_mir	29.501	BSP T0	26.591	54.403	BSP T1	25.885	9.627
HS	hsa-miR-653-5p-479134_mir	26.623	BSP T0	23.687	55.407	BSP T1	24.073	4.597
HS	hsa-miR-552-3p-479036_mir	31.08	BSP T0	28.059	56.672	BSP T1	29.473	2.36
HS	hsa-miR-645-478188_mir	29.52	BSP T0	26.497	56.784	BSP T1	27.738	2.664
HS	hsa-miR-490-3p-478131_mir	23.024	BSP T0	19.968	60.201	BSP T1	20.519	4.459
HS	hsa-miR-122-5p-477855_mir	26.108	BSP T0	23.044	60.548	BSP T1	24.032	3.312
HS	hsa-miR-548e-3p-478362_mir	31.255	BSP T0	28.027	65.425	BSP T1	28.567	4.99
HS	hsa-miR-133b-480871_mir	32.515	BSP T0	29.309	66.777	BSP T1	30.008	4.462
HS	hsa-miR-325-478025_mir	28.173	BSP T0	24.922	68.912	BSP T1	26.084	3.341
HS	hsa-miR-455-3p-478112_mir	33.736	BSP T0	30.328	76.847	BSP T1	30.469	7.559
HS	hsa-miR-562-479047_mir	33.3	BSP T0	29.774	80.494	BSP T1	31.22	3.276
**HS**	**hsa-miR-452-3p-478917_mir**	**35.433**	**BSP T0**	**31.841**	**84.227**	**BSP T1**	**31.853**	**9.261**
**HS**	**hsa-miR-27a-5p-477998_mir**	**32.41**	**BSP T0**	**28.776**	**86.698**	**BSP T1**	**29.712**	**5.028**
**HS**	**hsa-miR-302d-3p-478237_mir**	**35.322**	**BSP T0**	**31.685**	**86.877**	**BSP T1**	**33.221**	**3.322**
**HS**	**hsa-miR-483-5p-478432_mir**	**27.313**	**BSP T0**	**23.476**	**######**	**BSP T1**	**24.255**	**6.537**
**HS**	**hsa-miR-1-3p-477820_mir**	**23.639**	**BSP T0**	**19.302**	**######**	**BSP T1**	**21.532**	**3.382**
**HS**	**hsa-miR-193a-5p-477954_mir**	**28.946**	**BSP T0**	**24.264**	**######**	**BSP T1**	**25.798**	**6.958**
**HS**	**hsa-miR-34a-3p-478047_mir**	**36.784**	**BSP T0**	**30.723**	**######**	**BSP T1**	**34.673**	**3.347**
**HS**	**hsa-miR-1255a-478661_mir**	**34.043**	**BSP T0**	**27.562**	**######**	**BSP T1**	**26.948**	**######**
HS	hsa-let-7b-3p-478221_mir	27.175	BSP T0	-	-	BSP T1	25.057	3.364
HS	hsa-let-7f-5p-478578_mir	25.484	BSP T0	-	-	BSP T1	23.693	2.717
HS	hsa-let-7i-3p-477862_mir	30.612	BSP T0	-	-	BSP T1	29.239	2.007
HS	sa-miR-103a-2-5p-477864_m	27.283	BSP T0	-	-	BSP T1	23.675	9.572
HS	hsa-miR-1249-3p-478654_mir	26.405	BSP T0	-	-	BSP T1	27.582	0.343
HS	hsa-miR-130b-5p-477899_mir	27.354	BSP T0	-	-	BSP T1	25.699	2.439
HS	hsa-miR-132-3p-477900_mir	26.414	BSP T0	-	-	BSP T1	26.057	1.005
HS	hsa-miR-142-3p-477910_mir	26.847	BSP T0	-	-	BSP T1	25.704	1.734
HS	hsa-miR-143-3p-477912_mir	26.03	BSP T0	-	-	BSP T1	27.125	0.368
HS	hsa-miR-151b-477811_mir	22.894	BSP T0	-	-	BSP T1	21.253	2.416
HS	hsa-miR-154-3p-478725_mir	30.561	BSP T0	-	-	BSP T1	30.142	1.036
HS	hsa-miR-154-5p-477925_mir	32.164	BSP T0	-	-	BSP T1	30.329	2.8
HS	hsa-miR-193b-3p-478314_mir	28.743	BSP T0	-	-	BSP T1	26.92	2.777
HS	hsa-miR-20b-5p-477804_mir	26.436	BSP T0	-	-	BSP T1	27.077	0.503
HS	hsa-miR-216a-5p-477976_mir	28.771	BSP T0	-	-	BSP T1	27.619	1.745

HS	hsa-miR-296-5p-477836_mir	28.023	BSP T0	-	-	BSP T1	25.906	3.406
HS	hsa-miR-29a-3p-478587_mir	26.297	BSP T0	-	-	BSP T1	25.157	1.73
HS	hsa-miR-29b-3p-478369_mir	28.174	BSP T0	-	-	BSP T1	26.364	2.753
HS	hsa-miR-301a-3p-477815_mir	29.972	BSP T0	-	-	BSP T1	28.456	2.246
HS	hsa-miR-30b-5p-478007_mir	23.169	BSP T0	-	-	BSP T1	22.342	1.394
HS	hsa-miR-30c-5p-478008_mir	23.611	BSP T0	-	-	BSP T1	23.212	1.036
HS	hsa-miR-31-5p-478015_mir	29.062	BSP T0	-	-	BSP T1	32.663	0.065
HS	hsa-miR-345-5p-478366_mir	28.997	BSP T0	-	-	BSP T1	25.992	6.303
HS	hsa-miR-369-3p-478067_mir	28.786	BSP T0	-	-	BSP T1	26.893	2.916
HS	hsa-miR-374b-3p-479421_mir	35.145	BSP T0	-	-	BSP T1	32.741	4.102
HS	hsa-miR-432-5p-478101_mir	27.615	BSP T0	-	-	BSP T1	24.948	4.919
HS	hsa-miR-487b-3p-477835_mir	27.568	BSP T0	-	-	BSP T1	26.984	1.177
HS	hsa-miR-497-5p-478138_mir	29.314	BSP T0	-	-	BSP T1	27.124	3.534
HS	hsa-miR-518e-3p-479408_mir	32.903	BSP T0	-	-	BSP T1	30.637	3.778
HS	hsa-miR-574-3p-478163_mir	29.618	BSP T0	-	-	BSP T1	27.202	4.19
HS	hsa-miR-576-3p-478164_mir	25.626	BSP T0	-	-	BSP T1	25.544	0.831
HS	hsa-miR-606-479087_mir	28.136	BSP T0	-	-	BSP T1	28.729	0.514
HS	hsa-miR-183-3p-477936_mir	34.885	BSP T0	37.177	1,426	BSP T1	-	-
HS	hsa-miR-10b-5p-478494_mir	32.177	BSP T0	33.691	2,536	BSP T1	-	-
HS	hsa-miR-188-5p-477943_mir	31.115	BSP T0	31.24	6.408	BSP T1	-	-
HS	hsa-miR-383-5p-478079_mir	35.341	BSP T0	34.21	15.864	BSP T1	-	-
HS	hsa-miR-10b-3p-477868_mir	30.459	BSP T0	28.395	29.205	BSP T1	-	-
**HS**	**hsa-miR-188-3p-477942_mir**	**-**	**BSP T0**	**28.663**	**-**	BSP T1	**-**	**-**

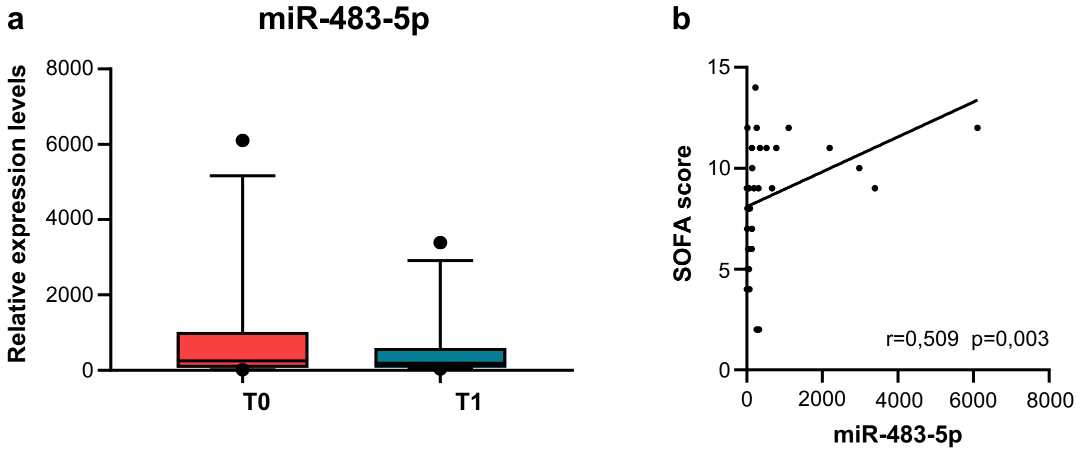

Figure S3. (**a**) Relative expression levels of miR-483-5p in plasma EVs of BSP T0 (n=18) and BSP T1 (n=18). (**b**) Correlation with miR-483-5p and SOFA score. Data are presented as median (IQR), normalized with a reference exogenous control (Cel-miR-39).

Table S3: Reagent information

Detection assay	Company	Code
hsa-miR-452-3p-478917	Life technologies	478917_mir
hsa-miR-27a-5p-477998	Life technologies	477998_mir
hsa-miR-302d-3p-478237	Life technologies	478237_mir
hsa-miR-483-5p-478432	Life technologies	478432_mir
hsa-miR-1-3p-477820	Life technologies	477820_mir
hsa-miR-193a-5p-477954	Life technologies	477954_mir
hsa-miR-34a-3p-478047	Life technologies	478047_mir
hsa-miR-1255a-478661	Life technologies	478661_mir
hsa-miR-188-3p-477942	Life technologies	477942_mir
Cel-miR-39-3p	Life technologies	478293_mir
